# The Hyperlipidemia Caused by Overuse of Glucocorticoid after Liver Transplantation and the Immune Adjustment Strategy

**DOI:** 10.1155/2017/3149426

**Published:** 2017-01-17

**Authors:** Xueqin Meng, Xinhua Chen, Liming Wu, Shusen Zheng

**Affiliations:** ^1^Collaborative Innovation Center for Diagnosis and Treatment of Infectious Diseases, The First Affiliated Hospital, Zhejiang University, Hangzhou 310003, China; ^2^Key Laboratory of Combined Multi-Organ Transplantation, Ministry of Public Health, The First Affiliated Hospital, Zhejiang University, Hangzhou 310003, China; ^3^The Department of Hepatobiliary and Pancreatic Surgery, The First Affiliated Hospital, Zhejiang University, Hangzhou 310003, China

## Abstract

The overuse of glucocorticoid may cause the metabolic disorders affecting the long term outcome of liver transplantation. This study aims to investigate the immune adjustment strategy by decreasing use of glucocorticoid after liver transplantation. The follow-up study was carried out on liver function and lipid metabolism. This study included adult recipients of liver transplantation. There were 3 groups according to their use of glucocorticoid: long term (>3 months, *n* = 18), short term (<3 months, *n* = 20), and control group (no use of glucocorticoid, radical hepatic resection, *n* = 22). The laboratory results of liver function (AST/ALT ratio) and serum lipid were compared 6 months after liver transplantation. AST/ALT ratio, the marker of liver function, showed no significant difference between long and short term group (*P* > 0.05). The acute rejection had no significant difference between short and long term groups, while TG, HDL, LDL, and glucose showed significant change in the long term group (*P* < 0.05). At 6 months after liver transplantation, the long term group showed higher metabolic disorders (*P* < 0.05). The proper immune adjustment strategy should be made to avoid overuse of glucocorticoid. It can decrease hyperlipidemia and other metabolic disorders after liver transplantation without increasing the acute rejection or liver function damage.

## 1. Introduction 

After liver transplantation, the overuse of glucocorticoid will cause glucocorticoid-related complications, such as new-onset diabetes, hypertension, osteoporosis, growth retardation, and hyperlipidemia [1]. With the application of new potent immune suppress agents, acute rejection after liver transplantation is not the main factor affecting the prognosis [2]. After raising the Hangzhou criteria, outcomes of liver transplant in our center continued to improve in recent decade. The liver transplantation recipients in 1-, 3-, and 5-year overall survival rate achieved 90.41%, 78.04%, and 72.45% [3]. The allograft-related perisurgery complications are decreasing. Prevention of hyperlipidemia and other metabolic disorders can decrease cardiovascular complications and improve the long term outcomes. Liver transplant is considered a coronary heart disease high risk based on the National Cholesterol Education Program Expert Panel on Detection, Evaluation, and Treatment of High Blood Cholesterol in Adults [4]. The study aims to compare the different use of glucocorticoid and the impact on liver function and hyperlipidemia after liver transplantation.

## 2. Materials and Methods

### 2.1. Patients

End-stage liver cirrhosis patients who had liver transplantation were followed up. Liver disease pathology was confirmed by laboratory examinations and liver biopsies. The primary disease, surgery procedure, gender, and age in different groups were matched. The study was approved by the Ethics Committee of the First Hospital of Zhejiang University. The details were present in Table 1. The adrenocorticotropic hormone (ACTH) has been tested and Cushing's syndrome has been excluded.

We follow up the adult liver transplantation recipients with the matched disease and surgeries. There were 3 groups according to their use of glucocorticoid: long term (>3 months, *n* = 18), short term (<3 months, *n* = 20), and control group (no use of glucocorticoid, radical hepatic resection, and *n* = 22). The laboratory results of liver function, serum lipid, and metabolic disorder occurrence were compared at 6 months after LT.

### 2.2. Immunosuppression Strategy

All transplantation recipients received immunosuppressants. The immunosuppressive medications (cyclosporine/corticosteroid) were in early stage after liver transplantation. We tried to cut off the time of corticosteroids within 3 months. 1000 mg prednisolone on the first day and 20 mg tapered to zero within the first 3 months.

### 2.3. Follow-Up Study

Adult recipients of liver transplantation were followed up according to the time of taking glucocorticoid: long term use of glucocorticoid (>3 month, *n* = 18), short term use of glucocorticoid (<3 month, *n* = 20), and control group (no use of glucocorticoid, radical hepatic resection, and *n* = 22).

### 2.4. Laboratory Tests

Laboratory results of liver function and metabolic function 6 months after LT were compared. Blood samples following 12 h fasting and 2 h after dinner were drawn from vein for adrenocorticotropic hormone (ACTH), cortisol, glucose, and serum lipids (total cholesterol, LDL, HDL, and triglyceride). ACTH was tested by electrochemiluminescence Elecsys ACTH immunoassay, ECLIIA, (Roche Diagnostics, Mannheim, Germany) on E170 Model. Cortisol was tested by a chemiluminescence assay (Bayer, Shanghai, China) on the ACS180 SE Model. The biochemistry exams were done by automated chemistry analyzer (Hitachi, Tokyo, Japan) on Hitachi 7600-110 Model. The liver function and lipids were tested by BECKMAN COULTER analyzer (Beckman Coulter Diagnostics, CA, USA) on AU5811 Model.

We measured the new onset diabetes according to the American Diabetes Association/World Health Organization guidelines; new onset DM was defined as patient used to have a normal fasting blood glucose, but after liver transplantation, he/she has a fasting blood glucose of  ≥7.00 mmol/L (1.26 g/L) confirmed on at least 2 occasions or current treatment with an oral antidiabetic drug or insulin. The pathological slides from the biopsy were parallel to the liver function results.

### 2.5. Statistical Analysis

Data were expressed as mean ± SD. For statistical comparison of values, Student's *t*-test was used by software SPSS 17.0. *P* values less than 0.05 were deemed to indicate statistical significance.

## 3. Results

### 3.1. AST/ALT Ratio Significantly Changed in Patients Who Took Glucocorticoid

**Figure 1:**
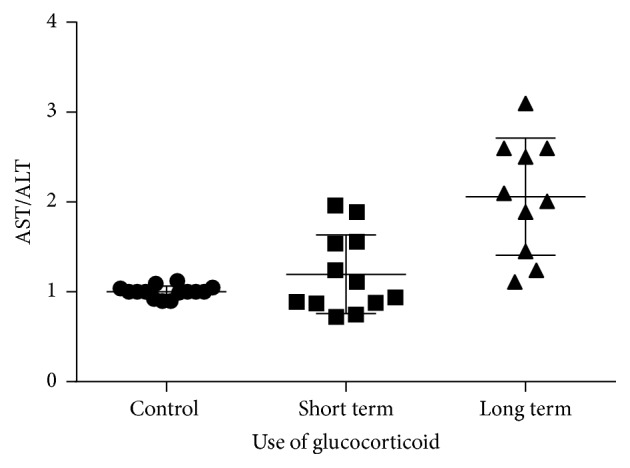
The AST/ALT ratio in patients taking short term, long term, and no glucocorticoid. Levels of AST/ALT ratio were significantly higher (*P* < 0.05) in patients who took glucocorticoid no matter long term or short term compared with control. There were no significant difference of AST between short or long term groups. Data are expressed in AST/ALT which is a marker to reflect liver function damage.

The 3 groups of patients who took glucocorticoid at different times after liver surgery were shown in Figure 1. There was no acute rejection in recipients, indicating that the use of glucocorticoid helped the transplanted liver to work properly and, as a results, patients survived. AST/ALT ratio had no significant difference between short or long term groups, which indicates that the short use of glucocorticoid less than 3 months can achieve the same immune effect as long term use of glucocorticoid longer than 3 months.

### 3.2. Cutting off the Overuse of Glucocorticoid Can Decrease TG

**Figure 2:**
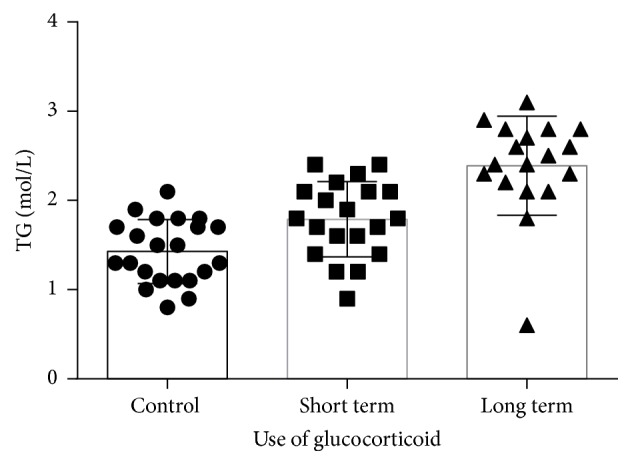
The TG levels in patients taking short term, long term, and no glucocorticoid. The quantitative evaluation of TG was analyzed and expressed in mol/L. TG significantly increased in patients who took different time of glucocorticoid compared with the control group. When comparing short term and long term use of glucocorticoid, short term group significantly decreased versus long term group (*P* < 0.05).

The quantitative evaluation of serum lipids was analyzed (Figure 2). TG significantly increased in patients who took different time of glucocorticoid compared with the control group (*P* < 0.05), indicating that the use of glucocorticoid can affect the lipid metabolism and lead to hyperlipidemia. When comparing short term and long term use of glucocorticoid, short term group significantly had decreased TG versus long term group (*P* < 0.05), indicating that cutting off the overuse of glucocorticoid can effectively decrease TG.

### 3.3. The Acute Rejection Occurrence

**Figure 3:**
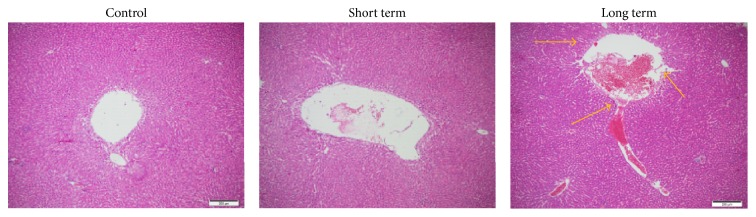
The histopathological results. The biopsy from patients in short term, long term, and control groups was shown. The short term indicates no significant difference from the control group while the long term group had sporadic inflammatory cells infiltration (the arrow indicates the lymphocytes). There was no obvious steatosis in any groups.

With development of surgery skill and organ protection technology, acute rejection becomes less common. All episodes of acute rejection in this study occurred in the first 6 weeks in recipients with a donor mismatches (Table 2). The prevalence of acute rejection was 10% in short term group versus 17% in long term group (*P* = 0.899364). Clinical symptoms were not typical. Laboratory tests on liver function were, for example, elevations AST (Figure 1). The diagnosis of acute rejection was confirmed by needle biopsy. The histopathological findings in reports from Department of Pathology showed that acute rejection includes predominantly mononuclear portal inflammation; subendothelial inflammation of portal and hepatic veins; and bile duct inflammation and damage. The short and long term groups had no significant difference in liver damage such as duct damages or unequivocal endotheliitis except some inflammatory infiltration in long term group. There were no visible steatosis cells in any groups (Figure 3).

### 3.4. The Metabolic Disorder Occurrence

Features of the metabolic disorders were found in patients 6 months after transplantation (Tables 2 and 3). New onset diabetes rose from 15% in short term group to 61% in long term group (*P* = 0.009174, Table 2); hypertension increased from 10% in short term group to 50% in long term group (*P* = 0.018447, Table 2). The long term group had significantly reduced HDL and increased LDL and 2 h after dinner blood glucose (Table 3).

### 3.5. The Occurrence of Glucocorticoid-Related Side Effects

Occurrence of glucocorticoid-related side effects such as infection and poor wound healing was found in patients followed up >6 months after transplantation (Table 2). The prevalence of infection rises from 15% in short term group to 56% in long term group (*P* = 0.022093). Poor wound healing increased from 10% in short term group to 39% in long term group (*P* = 0.087380). The reinfection with hepatitis B and the infection with CMV have been excluded.

## 4. Discussion 

Lipid metabolic disorders are becoming more common after liver transplantation which affect the long term morbidity and mortality as well as life quality. Some patients do have the metabolic preconditions such as overweight, diabetes, and cholestatic liver disease. The new onset of metabolic disorders is often caused by the use of immunosuppressant medications such as cyclosporine, tacrolimus, and glucocorticoid which attack the insulin signaling pathway by changing adipose enzymes [5].

We monitor patients' liver function and lipid levels 6 months after transplant. The results indicate that avoiding overuse of glucocorticoid can significantly decrease the risk of hyperlipidemia in liver transplant recipients.

It has been well known that glucocorticoid inhibits immune function. The metabolic side effects have been noticed recently such as diabetes, hypertension, and hyperlipidemia [6].

Because acute rejection after liver transplantation generally occurred 2 weeks after liver transplantation, we suggest early glucocorticoid withdrawal scheme to ensure a lower rejection and minimize the amount of glucocorticoid. Our clinical data suggest that the minimized use of glucocorticoid did not increase the incidence of rejection but decreased postoperative infection and poor wound healing. Quite different from some liver transplantation centers whose primary liver diseases of liver transplantation are alcoholic cirrhosis, HCV cirrhosis, and autoimmune liver disease, our center has more than 90% of liver transplant recipients for HBV-related diseases, with about 40% of hepatocellular carcinoma. Overuse of glucocorticoid after liver transplantation for more than 1 year also causes the cancer and HBV recurrence [7, 8].

The blood glucose changes caused by the long term use of steroid are different from type 1 and type 2 diabetes in terms of etiology, clinical features, and treatment strategies. One unique finding is that the glucocorticoid-induced blood glucose changes on fasting and randomly were different. Because glucocorticoids were administered at 8:00 am, these patients' fasting glucose was not affected. But the 2 h blood glucose after dinner significantly increased compared to control group.

Steroids have been used for the purpose of preventing rejection. However, steroid-related metabolic disorders, such as hypertension, diabetes mellitus, hyperlipidemia, and obesity, are causing concerns for its long term use. Hyperlipidemia significantly increases cardiovascular events. In addition, the hyperlipidemia caused atherosclerosis, which can reduce liver perfusion, resulting in deterioration of liver function. Hyperlipidemia also participates in the chronic graft rejection similar to the proliferate vascular disease.

Our result demonstrated that a short term use of steroid (<3 months) was safe in patients undergoing LT for HBV-related HCC. Liver function recovered significantly better than those of long term use of steroid (>3 months).

The patients who had liver transplantation for their autoimmune hepatitis do have difficulties stopping steroid because the high chance of immune hepatitis recurrence. There are a few patients who need to use steroid because of rejection. When the acute rejection occurs, high dose steroid is still the first line medicine. Other than the occasions mentioned above, the majority of adult recipients can stop steroid safely within 3 months. Previous study [9] concluded that steroid tapering to 5 mg/day does not lead to graft loss and may decrease the incidence and severity of late metabolic complications. When there is no immune hepatitis nor acute rejection, the steroid can be discontinued 14 d after liver transplantation when immunosuppressive therapy based on FKS06+ MMF/CsA was still maintained [10, 11].

## 5. Conclusion 

Glucocorticoid after liver transplantation is one of the most commonly used immunosuppressants, playing a very important role in the prevention and treatment of acute rejection. However, long term use of glucocorticoid has many side effects. Early withdraw within 3 months can decrease the risk of metabolic disorders.

## Figures and Tables

**Table 1:** The baseline and characteristics of patients.

	Control without glucocorticoid	Short term use of glucocorticoid	Long term use of glucocorticoid
Number of patients	22	20	18
Time of glucocorticoid	0	<3 months	>3 months
Gender (male/female)	10/12	11/10	10/8
Recipient age	52 ± 8.3	53 ± 7.4	48 ± 5.1
MELD score	16.3 ± 4.1	24.9 ± 7.3	22.9 ± 6.7
Surgery	Hepatic resection	Orthotopic liver transplantation	Orthotopic liver transplantation
CSA	No	Yes	Yes
BMI	24 ± 3.4	26 ± 1.4	28 ± 1.1
Basic diseases	Angioma	Liver cirrhosis	Liver cirrhosis

**Table 2:** The complications in patients with use of glucocorticoid after liver transplantation.

	Infection	Wound nonhealing	New onset diabetes	Hypertension	Acute rejection
Short term	3	2	3	2	2
Long term	10	7	11	9	3
*χ* ^2^	5.238479	6.788542	5.553217	5.553217	0.015993
*P*	0.022093	0.009174	0.018447	0.018447	0.899364

**Table 3:** The baseline and characteristics of patients.

	Control without glucocorticoid	Short term use of glucocorticoid	Long term use of glucocorticoid
Number of patients	22	20	18
Time of glucocorticoid	0	<3 months	>3 months
Glucose fasting (mmol/L)	4.9 ± 1.3	5.1 ± 0.7	5.3 ± 1.1
Glucose 2 h after dinner (mmol/L)	6.9 ± 0.7	7.1 ± 0.7	9.3 ± 2.6^*∗*^
ACTH 8 am (pmol/L)	6.5 ± 0.4	7.2 ± 0.7	7.9 ± 0.3
HDL (high density lipoprotein, mmol/L)	1.49 ± 0.31	1.29 ± 0.24	0.99 ± 0.21^*∗*^
LDL (low density lipoprotein, mmol/L)	2.29 ± 0.81	2.71 ± 0.53	3.09 ± 0.74^*∗*^
TC (total cholesterol, mmol/L)	5.29 ± 0.37	5.61 ± 0.21	5.78 ± 0.52

^*∗*^ *P* < 0.05 versus control group.
